# Association of the Triglyceride–Glucose (TyG) Index and TyG‐BMI With 1‐Year Outcomes Following Endovascular Treatment in Acute Basilar Artery Occlusion

**DOI:** 10.1002/cns.70898

**Published:** 2026-04-23

**Authors:** Qiuting Wang, Haodong Li, Yuxin Chen, Na Liu, Lu Yang, Chuanjie Wu, Wenbo Zhao, Longfei Wu, Liqun Jiao, Sijie Li, Qingfeng Ma, Xunming Ji, Chuanhui Li

**Affiliations:** ^1^ Department of Neurology Xuanwu Hospital, Capital Medical University Beijing China; ^2^ Department of Neurosurgery Xuanwu Hospital, Capital Medical University Beijing China

**Keywords:** basilar artery, insulin resistance, mortality, thrombectomy

## Abstract

**Background:**

Long‐term outcomes after endovascular treatment (EVT) in acute basilar artery occlusion (BAO) vary substantially, yet metabolic correlates remain incompletely characterized. This study evaluated whether the triglyceride–glucose (TyG) index and TyG–body mass index (TyG‐BMI) are associated with 1‐year functional status and mortality after EVT.

**Methods:**

A single‐center registry with prospective data collection was retrospectively analyzed. Consecutive acute BAO patients undergoing EVT within 24 h (December 2012–September 2024) were included. Unfavorable functional outcome at 1 year was defined as modified Rankin Scale (mRS) 4–6; 1‐year all‐cause mortality was also assessed. Multivariable logistic regression was applied, with receiver operating characteristic analyses and prespecified subgroup analyses.

**Results:**

The final cohort comprised 298 patients (median age 62 years; 78.5% male), among whom 165 (55.4%) had an unfavorable 1‐year outcome and 109 (36.6%) died. After adjustment, each 1‐SD increase in TyG and TyG‐BMI corresponded to higher odds of unfavorable outcome (TyG OR 1.69, 95% CI 1.25–2.30; TyG‐BMI OR 1.46, 95% CI 1.10–1.96) as well as mortality (TyG OR 1.44, 95% CI 1.09–1.90; TyG‐BMI OR 1.61, 95% CI 1.23–2.12). Discrimination was modest and similar between indices. A significant sex interaction was observed for TyG‐BMI and mortality (*p* for interaction = 0.03), with an association in men only.

**Conclusions:**

In EVT‐treated acute BAO, TyG and TyG‐BMI may help identify EVT‐treated acute BAO patients at higher risk of poor 1‐year outcome and death. The mortality risk associated with TyG‐BMI was modified by sex, with an effect confined to men.

## Graphical abstract

TyG and TyG‐BMI independently predicted unfavorable functional outcome and 1‐year mortality after endovascular treatment for acute basilar artery occlusion. Higher values were associated with worse prognosis, with TyG‐BMI showing a sex‐specific effect on mortality, confined to men.

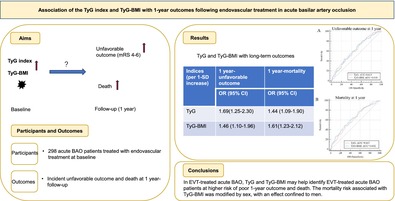

## 1 Introduction

Acute basilar artery occlusion (BAO) is a high‐risk cerebrovascular emergency, often resulting in severe disability or death [1]. Endovascular treatment (EVT) has become a key reperfusion strategy for posterior circulation large vessel occlusion (LVO), and several randomized controlled trials in recent years have supported its efficacy in selected patients with acute BAO [2]. Nevertheless, long‐term functional recovery remains highly variable even after technically successful recanalization, highlighting the need for readily available prognostic markers.

Insulin resistance (IR) has been implicated in poorer cerebrovascular outcomes. The triglyceride–glucose (TyG) index, calculated from routine fasting triglyceride and glucose values, is commonly used as a practical indicator of this metabolic state [3, 4]. In acute ischemic stroke, higher TyG has been linked to less favorable short‐term outcomes across multiple cohorts, including patients treated with reperfusion approaches such as intravenous thrombolysis (IVT) and EVT, where functional status is often evaluated at 90 days [5, 6, 7, 8]. To better capture the metabolic interplay between dyslipidemia and adiposity, the TyG–body mass index (TyG‐BMI) has also been evaluated [9, 10]. However, findings have been heterogeneous across stroke populations, with some reports suggesting paradoxical or inconsistent associations with short‐term functional outcomes [11, 12]. Importantly, evidence on long‐term endpoints is limited, leaving uncertainty about whether TyG and TyG‐BMI relate to longer‐term prognosis in acute BAO patients undergoing EVT.

Therefore, in this single‐center retrospective cohort of EVT‐treated acute BAO, the relationships of TyG and TyG‐BMI with 1‐year unfavorable functional outcome and 1‐year mortality were assessed. In addition, mRS distributions across tertiles were examined, exposure–response patterns were characterized through restricted cubic spline (RCS) analyses, discriminatory performance was evaluated using receiver operating characteristic (ROC) analyses, and prespecified subgroup analyses were conducted to evaluate potential effect modification.

## 2 Methods

### 2.1 Study Design

**FIGURE 1:**
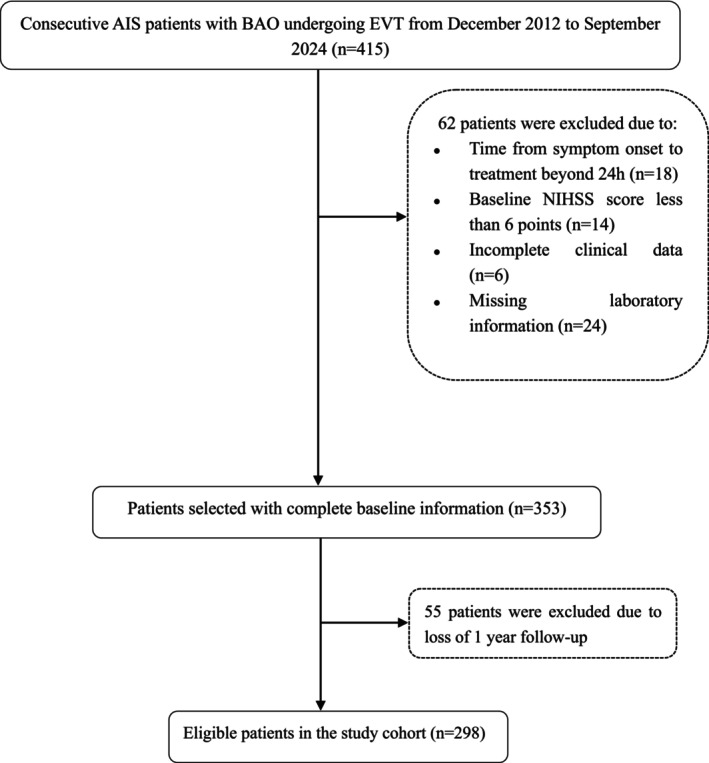
Flowchart of patients included in this study. AIS, acute ischemic stroke; BAO, basilar artery occlusion; EVT, endovascular treatment; NIHSS, National Institutes of Health Stroke Scale.

This study retrospectively analyzed an institutional BAO registry at Xuanwu Hospital, Capital Medical University, which has been prospectively maintained [13]. Consecutive records from December 2012 to September 2024 were screened, and the analytic cohort was assembled according to prespecified eligibility criteria (Figure 1).

Briefly, among consecutive EVT‐treated acute BAO patients (*n* = 415), patients were excluded if EVT was initiated more than 24 h after symptom onset or last known well (LKW), clinical data were incomplete, a baseline National Institutes of Health Stroke Scale (NIHSS) score was less than 6, or laboratory data were missing. This yielded 353 patients with complete baseline information. After excluding patients without 1‐year follow‐up, 298 participants remained for the final analysis.

### 2.2 Data Collection

Baseline demographic and clinical information was obtained in a standardized manner, including vascular risk factors and pre‐stroke medication use. Stroke severity at presentation was quantified with the NIHSS [14], and consciousness level was evaluated by using the Glasgow Coma Scale (GCS). Premorbid functional status was recorded by the modified Rankin Scale (mRS). Baseline imaging severity in the posterior circulation was rated by the posterior circulation Alberta Stroke Program Early Computed Tomography Score (pc‐ASPECTS) [15]. Stroke etiology was classified according to established ischemic stroke subtype criteria [16]. Baseline fasting laboratory values were retrieved from electronic medical records, including triglycerides (TG), fasting plasma glucose (FPG), and total cholesterol. Treatment‐ and procedure‐related variables comprised IVT, tirofiban use, onset/LKW‐to‐groin puncture and onset/LKW‐to‐reperfusion time intervals, anesthesia type, and procedural details. Angiographic reperfusion was graded according to the modified Thrombolysis in Cerebral Infarction (mTICI) scale, and mTICI ≥ 2b indicated successful reperfusion [17].

### 2.3 Measurement of TyG and TyG‐BMI

The TyG and TyG‐BMI indices were calculated by using the established formulas shown below [18]:

$$\mathrm{TyG}=\ln\;\left[\frac{\mathrm{TG}\;\left(\mathrm{mg}/\mathrm{dL}\right)\;X\;\mathrm{FPG}\;\left(\mathrm{mg}/\mathrm{dL}\right)}{2}\right]$$

TyG−BMI=TyGxBMI

### 2.4 Outcomes Assessments

Unfavorable functional status at 1 year was operationalized as an mRS score of 4–6, and 1‐year all‐cause death was additionally recorded [19]. Functional status at 1 year was obtained from clinic records or structured telephone follow‐up with patients/relatives, supplemented by medical record verification. In‐hospital safety outcomes comprised symptomatic intracranial hemorrhage (sICH) and procedure‐related complications, along with other serious adverse events. Intracranial hemorrhage was classified as symptomatic if accompanied by a ≥ 4‐point worsening in NIHSS or death within 24 h [20].

### 2.5 Statistical Analysis

Missing data were handled using a complete‐case approach. Only participants with complete data for the exposure, outcomes, and all covariates of interest were included; therefore, all analyses were performed in the same complete‐case dataset and no imputation was conducted. Summary measures were selected based on data distribution. Continuous variables are reported as mean (standard deviation, SD) for approximately normal data and as median (interquartile range) for skewed data, whereas categorical variables are presented as number (percentage). Between‐group comparisons used Student's *t*‐test for normally distributed continuous variables and the Mann–Whitney *U* test otherwise. Categorical data were compared using the chi‐square test or Fisher's exact test, as appropriate.

Multivariable logistic regression was used to evaluate how TyG and TyG‐BMI relate to clinical outcomes, with effect sizes summarized as odds ratios (ORs) and 95% confidence intervals (CIs). Analyses treated TyG and TyG‐BMI as continuous measures (per 1‐SD increment) and as tertiles. Discrimination performance was evaluated by ROC curves with area under the curve (AUC) comparisons. Possible nonlinear patterns were explored using RCS modeling. Effect modification was assessed by adding interaction terms between TyG or TyG‐BMI and prespecified demographic and clinical factors. When a significant interaction was identified, stratified analyses were performed to characterize associations within subgroups. A two‐sided *p* value < 0.05 was considered statistically significant. Statistical analyses were performed by using IBM SPSS Statistics (version 27.0), RStudio (version 4.2.3), and MedCalc (version 20.100).

## 3 Results

### 3.1 Baseline Characteristics

In the final cohort (*n* = 298), 133 patients (44.6%) achieved a favorable 1‐year functional outcome and 165 (55.4%) had an unfavorable outcome. Overall, the median age was 62 years and 234 patients (78.5%) were men. Baseline demographics and most vascular risk factors were largely comparable between groups (Table 1). Relative to patients with favorable outcomes, those with unfavorable outcomes more commonly had atrial fibrillation, prior ischemic stroke, and pre‐stroke antiplatelet use (all *p* < 0.05). They also presented with more severe deficits and lower consciousness, with slightly higher premorbid mRS scores. Laboratory measures differed between outcome groups, with FPG, TyG, and TyG‐BMI higher in those with unfavorable outcomes (all *p* < 0.01). Procedural characteristics also varied: tirofiban was used less often (*p* = 0.022), whereas general anesthesia (*p* = 0.003) and stent retriever deployment (*p* = 0.049) were more frequent, accompanied by a lower rate of successful reperfusion (*p* = 0.032).

**TABLE 1:** Baseline characteristics of study participants.

Characteristics	Total sample (*n* = 298)	Favorable outcome (*n* = 133)	Unfavorable outcome (*n* = 165)	*p*
Demographics				
Age (years)	62 (55–68)	61 (55–67)	63 (54–69.5)	0.178
Male, *n* (%)	234 (78.5)	105 (78.9)	129 (78.2)	0.873
BMI (kg/m^2^)	26.02 (24.22–27.76)	25.71 (24.22–27.68)	26.35 (24.33–28.20)	0.052
Medical history, *n* (%)				
Hypertension	244 (81.9)	110 (82.7)	134 (81.2)	0.739
Diabetes mellitus	93 (31.2)	37 (27.8)	56 (33.9)	0.257
Hyperlipidemia	118 (39.6)	58 (43.6)	60 (36.4)	0.204
Atrial fibrillation	50 (16.8)	16 (12)	34 (20.6)	0.049
Coronary heart disease	51 (17.1)	21 (15.8)	30 (18.2)	0.586
Ischemic stroke	97 (32.6)	33 (24.8)	64 (38.8)	0.010
Smoking	134 (45)	54 (40.6)	80 (48.5)	0.174
Drinking	114 (38.3)	50 (37.6)	64 (38.8)	0.833
Use of antiplatelet drugs	84 (28.2)	26 (19.5)	58 (35.2)	0.003
Use of anticoagulant drugs	10 (3.4)	6 (4.5)	4 (2.4)	0.350
Use of hypolipidemic drugs	48 (16.1)	24 (18)	24 (14.5)	0.414
Use of hypoglycemic drugs	70 (23.5)	30 (22.6)	40 (24.2)	0.733
Use of antihypertensive drugs	192 (64.4)	78 (58.6)	114 (69.1)	0.061
NIHSS on admission	23 (15–34)	17 (13–29)	27 (19.5–37)	< 0.001
pc‐ASPECTS	8 (7–9.25)	8 (7–10)	8 (7–9)	0.121
Premorbid mRS	0 (0–0)	0 (0–0)	0 (0–0.5)	0.012
GCS	7 (4–11)	10 (6–12.5)	6 (4–9.5)	< 0.001
Blood pressure on admission				
SBP	153.56 (24.30)	153.17 (22.93)	153.87 (25.41)	0.803
DBP	86 (77–96)	86 (76.5–96)	86 (77.5–96)	0.866
Stroke etiology, *n* (%)				0.428
Large vessel atherosclerosis	246 (82.6)	114 (85.7)	132 (80)	
Cardioembolic	46 (15.4)	17 (12.8)	29 (17.6)	
Other causes	6 (2)	2 (1.5)	4 (2.4)	
IVT, *n* (%)	65 (21.8)	32 (24.1)	33 (20)	0.399
Tirofiban treatment, *n* (%)	218 (73.2)	106 (79.7)	112 (67.9)	0.022
Time intervals, min				
OTP	490 (365–740)	522 (376.5–825)	478 (352–732.5)	0.155
OTR	573.5 (449–853.5)	585 (456.5–892.5)	570 (444.5–790.5)	0.320
General anesthesia, *n* (%)	150 (50.3)	54 (40.6)	96 (58.2)	0.003
Interventional procedures, *n* (%)				
Stent retriever	195 (65.4)	79 (59.4)	116 (70.3)	0.049
Aspiration	175 (58.7)	82 (61.7)	93 (56.4)	0.356
Intra‐arterial thrombolysis	44 (14.8)	20 (15)	24 (14.5)	0.905
Stenting	104 (34.9)	52 (39.1)	52 (31.5)	0.172
Balloon angioplasty	110 (36.9)	46 (34.6)	64 (38.8)	0.455
mTICI 2b‐3, *n* (%)	282 (94.6)	130 (97.7)	152 (92.1)	0.032
Laboratory parameters				
Fasting glucose, mg/dL	135.78 (111.50–184.16)	120.19 (102.17–154.52)	149.75 (121.82–208.13)	< 0.001
Total cholesterol, mg/dL	162.41 (135.64–188.90)	161.25 (136.51–195.09)	163.57 (134.77–188.13)	0.799
TG, mg/dL	100.06 (71.5–150.75)	97.40 (65.97–141.67)	104.48 (75.26–159.82)	0.090
TyG	8.83 (8.43–9.40)	8.73 (8.28–9.17)	8.95 (8.57–9.57)	< 0.001
TyG‐BMI	234.37 (39.51)	226.12 (39.02)	241.02 (38.75)	0.001

*Note:* Data were presented as number (%), median (interquartile range) or mean (±standard deviation).

Abbreviations: BMI, body mass index; DBP, diastolic blood pressure; GCS, Glasgow Coma Scale; IVT, Intravenous thrombolysis; mTICI, modified Thrombolysis in Cerebral Infarction; NIHSS, National Institutes of Health Stroke Scale; OTP, onset‐to‐puncture; OTR, onset‐to‐reperfusion; pc‐ASPECTS, posterior circulation Alberta Stroke Program Early Computed Tomography Score; SBP, systolic blood pressure; TyG‐BMI, triglyceride–glucose‐body mass index; TG, triglycerides; TyG index, triglyceride–glucose index.

Table S1 provides baseline comparisons by 1‐year survival. Non‐survivors had higher BMI (p = 0.003), higher baseline NIHSS score and lower GCS (both p < 0.001), higher premorbid mRS (p = 0.011), less frequent tirofiban use (p = 0.018), more frequent stent retriever use (p = 0.028), lower successful recanalization rates (p = 0.027), and higher FPG, TyG, and TyG‐BMI (all p < 0.001).

### 3.2 Association Between TyG and TyG‐BMI and Outcomes

**FIGURE 2:**
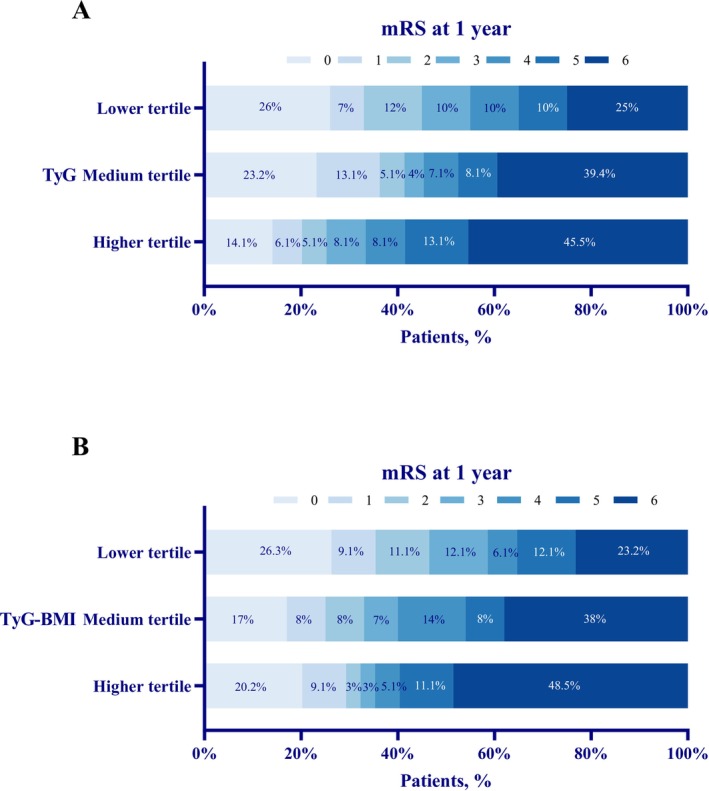
Distribution of 1‐year mRS scores across tertiles of TyG and TyG‐BMI. Significant differences in the distribution of 1‐year mRS scores were observed across TyG tertiles (Figure 2A; *p* = 0.043) and across TyG‐BMI tertiles (Figure 2B; *p* = 0.007). mRS, modified Rankin Scale; TyG index, triglyceride–glucose index; TyG‐BMI, triglyceride–glucose–body mass index.

The 1‐year mRS score distribution stratified by tertiles of TyG and TyG‐BMI is presented in Figure 2. Among the 298 included patients, 165 (55.4%) had an unfavorable 1‐year functional outcome and 109 (36.6%) died. Event rates increased across TyG tertiles: the highest tertile was associated with a greater proportion of unfavorable outcomes (66.7% vs. 45.0%, *p* = 0.009) and deaths (45.5% vs. 25.0%, *p* = 0.009) than the lowest tertile. A similar gradient was observed for TyG‐BMI, with higher rates of unfavorable outcome (64.6% vs. 41.4%, *p* = 0.002) and mortality (48.5% vs. 23.2%, *p* = 0.001) in the highest versus lowest tertile. Serious adverse events during hospitalization were largely comparable across TyG tertiles (Table S2). Across TyG‐BMI tertiles, brain herniation differed significantly (*p* = 0.006) and sICH showed a borderline difference (*p* = 0.056), whereas other events were similar (Table S3).

After multivariable adjustment (Table 2), higher TyG and TyG‐BMI were linked to greater odds of 1‐year unfavorable functional outcome and 1‐year mortality. Per 1‐SD increase, the adjusted ORs were 1.69 (95% CI 1.25–2.30) for TyG and 1.46 (1.10–1.96) for TyG‐BMI with unfavorable outcome; corresponding estimates for mortality were 1.44 (1.09–1.90) and 1.61 (1.23–2.12), respectively. Analyses using tertiles showed a similar pattern for unfavorable outcome, whereas the TyG–mortality association was attenuated and became nonsignificant after adjustment (Table 2).

**TABLE 2:** Associations of TyG and TyG‐BMI with long‐term outcomes.

Index	No. of participants	No. of cases	Univariate analysis		Multivariate analysis	
			OR (95% CI)	*p*	OR (95% CI)	*p*
1 year unfavorable outcome^a^						
TyG, per 1‐SD increase	298	165	1.62 (1.26–2.09)	< 0.001	1.69 (1.25–2.30)	< 0.001
TyG, categorical variable (tertiles)						
Lower	100	45	1.00 (reference)		1.00 (reference)	
Medium	99	54	1.47 (0.84–2.56)	0.179	1.54 (0.80–2.99)	0.200
Upper	99	66	2.44 (1.38–4.34)	0.002	2.26 (1.13–4.53)	0.021
*p* for linear trend				0.002		0.023
TyG‐BMI, per 1‐SD increase	298	165	1.48 (1.16–1.89)	0.001	1.46 (1.10–1.96)	0.010
TyG‐BMI, categorical variable (tertiles)						
Lower	99	41	1.00 (reference)		1.00 (reference)	
Medium	100	60	2.12 (1.21–3.74)	0.009	2.28 (1.17–4.44)	0.015
Upper	99	64	2.59 (1.46–4.59)	0.001	2.80 (1.41–5.57)	0.003
*p* for linear trend				0.001		0.003
1 year mortality^b^						
TyG, per 1‐SD increase	298	109	1.57 (1.23–2.02)	< 0.001	1.44 (1.09–1.90)	0.011
TyG, categorical variable (tertiles)						
Lower	100	25	1.00 (reference)		1.00 (reference)	
Medium	99	39	1.95 (1.06–3.58)	0.031	1.83 (0.92–3.64)	0.086
Upper	99	45	2.50 (1.37–4.56)	0.003	1.75 (0.88–3.49)	0.113
*p* for linear trend				0.004		0.170
TyG‐BMI, per 1‐SD increase	298	109	1.61 (1.25–2.06)	< 0.001	1.61 (1.23–2.12)	< 0.001
TyG‐BMI, categorical variable (tertiles)						
Lower	99	23	1.00 (reference)		1.00 (reference)	
Medium	100	38	2.03 (1.09–3.75)	0.025	2.06 (1.04–4.08)	0.039
Upper	99	48	3.11 (1.69–5.73)	< 0.001	3.11 (1.58–6.12)	0.001
*p* for linear trend				< 0.001		0.001

*Note:* Univariate and multivariate ORs with 95% CIs were obtained using logistic regression. Variables included in multivariate models were selected based on baseline comparisons for each endpoint (*p* < 0.10, Table 1, and Table S1) and entered for adjustment accordingly. For TyG‐BMI, BMI was not additionally adjusted in multivariate models because BMI is incorporated into the TyG‐BMI index.

Abbreviations: CI, confidence interval; GCS, Glasgow Coma Scale; mRS, modified Rankin Scale; NIHSS, National Institutes of Health Stroke Scale; OR, odds ratio; OTP, onset‐to‐puncture time; TyG‐BMI, triglyceride–glucose–body mass index; TyG index, triglyceride–glucose index.

^a^ For 1‐year unfavorable functional outcome, the multivariate model was adjusted for BMI, atrial fibrillation, ischemic stroke, use of antiplatelet drugs, use of antihypertensive drugs, NIHSS on admission, premorbid mRS, GCS, tirofiban treatment, general anesthesia, stent retriever use, and successful recanalization.

^b^ For 1‐year mortality, the multivariate model was adjusted for BMI, use of antiplatelet drugs, use of anticoagulant drugs, NIHSS on admission, premorbid mRS, GCS, tirofiban treatment, OTP, stent retriever use, and successful recanalization.

**FIGURE 3:**
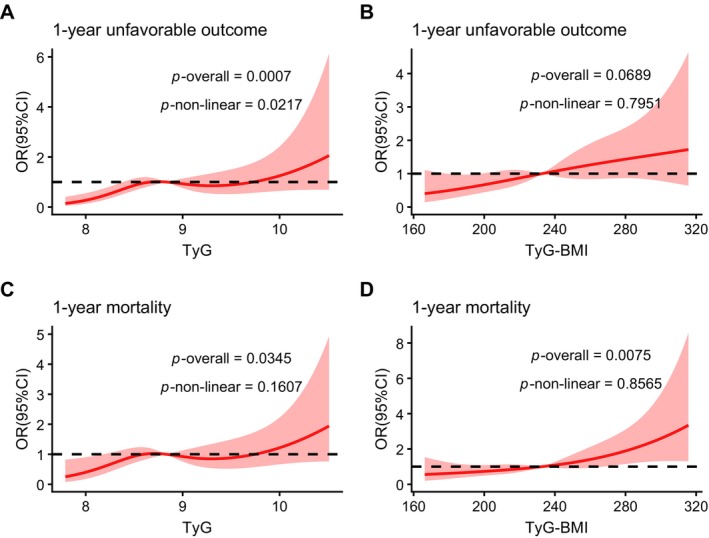
Restricted cubic spline analyses of TyG and TyG‐BMI in relation to 1‐year outcomes. Restricted cubic spline curves showing the adjusted associations of TyG and TyG‐BMI with 1‐year unfavorable functional outcome and 1‐year mortality. Panels show: (A) TyG and 1‐year unfavorable outcome; (B) TyG‐BMI and 1‐year unfavorable outcome; (C) TyG and 1‐year mortality; (D) TyG‐BMI and 1‐year mortality. Models were adjusted for the same covariates as in the multivariable analyses (Table 2); BMI was not included in TyG‐BMI models. The *p* values for the overall association (*p*‐overall) and nonlinearity (*p*‐nonlinear) are shown in each panel. CI, confidence interval; OR, odds ratio; BMI, body mass index; TyG index, triglyceride–glucose index; TyG‐BMI, triglyceride–glucose–body mass index.

Discrimination for 1‐year unfavorable outcome was modest for both indices (TyG AUC 0.619 vs. TyG‐BMI AUC 0.610; *p* = 0.7098). For 1‐year mortality, AUCs for TyG and TyG‐BMI were 0.617 and 0.632, respectively (*p* = 0.5790, Figure S1). RCS analyses were then used to characterize dose–response patterns (Figure 3). The spline model supported a non‐linear relationship between TyG and 1‐year unfavorable functional outcome, whereas TyG‐BMI showed only a borderline association. For 1‐year mortality, the relationships for both TyG and TyG‐BMI appeared approximately linear (Figure 3).

### 3.3 Subgroup Analyses

Across prespecified subgroups, the associations of TyG and TyG‐BMI with 1‐year unfavorable functional outcome, and the association of TyG with 1‐year mortality, remained broadly stable, with no statistically meaningful interactions (all *p* for interaction > 0.05; Figures S2–S4).

**FIGURE 4:**
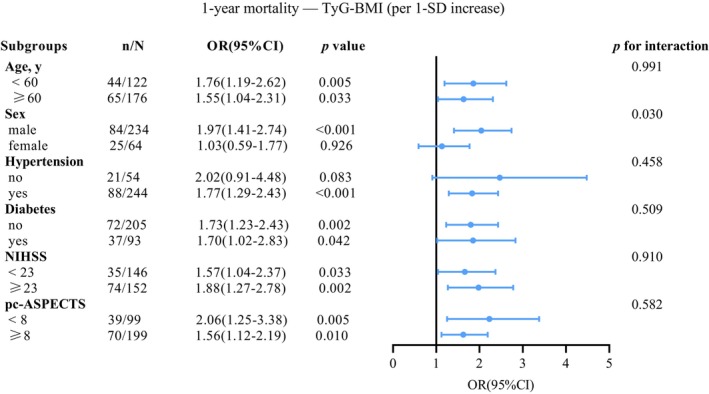
Subgroup analyses of the association between TyG‐BMI and 1‐year mortality. Forest plot of adjusted ORs for 1‐year mortality per 1‐SD increase in TyG‐BMI across prespecified subgroups. Points indicate ORs and horizontal lines indicate 95% CIs; the vertical line denotes OR = 1. A significant interaction was observed for sex (*p* for interaction = 0.030), whereas no significant interactions were detected for other subgroups. Adjustments were consistent with the primary multivariable model (Table 2). CI, confidence interval; OR, odds ratio; SD, standard deviation; TyG‐BMI, triglyceride–glucose–body mass index.

A different pattern emerged for TyG‐BMI in relation to 1‐year mortality: a significant interaction with sex was identified (*p* for interaction = 0.030, Figure 4), with an association evident among men but not among women. For other prespecified factors, interaction testing did not indicate heterogeneity (all *p* for interaction > 0.05, Figure 4).

## 4 Discussion

In this retrospective single‐center cohort of consecutive patients with acute BAO undergoing EVT, higher TyG and TyG‐BMI were independently associated with both 1‐year unfavorable functional outcome and 1‐year mortality. Subgroup analyses identified a significant interaction by sex for the association between TyG‐BMI and 1‐year mortality, which was evident in men but not in women. The ROC analyses suggested comparable discriminatory performance between TyG and TyG‐BMI for both outcomes. However, the observed AUC values indicate only modest discrimination, suggesting that TyG and TyG‐BMI have limited standalone predictive capability. Therefore, these indices should be interpreted primarily as associative markers that may support risk stratification rather than as definitive prognostic classifiers.

This study extends prior evidence linking IR‐related indices to poststroke outcomes. Most published work has examined TyG in broader acute ischemic stroke populations or in reperfusion‐treated cohorts using short‐term endpoints, typically 90‐day functional outcome, and the reported associations have been inconsistent across populations and treatment strategies [5, 6, 7, 8, 12, 21, 22]. Evidence for long‐term prognosis remains scarce, and to date only one EVT‐focused study has evaluated TyG‐related indices in relation to 1‐year functional outcome. In that cohort, TyG‐BMI was associated with 1‐year unfavorable outcome, which is consistent with the present findings, whereas TyG itself was not statistically associated with 12‐month outcome [12]. The discrepant result for TyG may be partly explained by differences in outcome definitions and case mix, because that study defined favorable outcome as mRS 0–2 at 12 months and was predominantly composed of anterior circulation LVO, with BAO accounting for only a small proportion of cases. Importantly, prior studies have not specifically focused on BAO to report associations of TyG and TyG‐BMI with 1‐year unfavorable functional outcome and 1‐year mortality, and the present findings therefore provide additional evidence that IR‐related metabolic burden is associated with long‐term disability and survival in this high‐risk population.

Several mechanisms may plausibly explain the observed associations. IR is linked to endothelial dysfunction, systemic inflammation, oxidative stress, and a prothrombotic milieu, which can contribute to microvascular no‐reflow and attenuated tissue reperfusion even after angiographic recanalization [23, 24]. These pathophysiological processes may impair blood–brain barrier integrity and contribute to edema formation and hemorrhagic transformation, which may adversely affect long‐term functional status and survival [25, 26]. The largely comparable in‐hospital adverse event profile across TyG tertiles and the signals across TyG‐BMI tertiles for brain herniation and borderline differences in sICH are compatible with a secondary injury pathway, although the low event counts warrant cautious interpretation.

The subgroup finding indicated that the association between TyG‐BMI and 1‐year mortality was evident in men but not in women, suggesting that adiposity‐related metabolic burden may have different prognostic implications by sex. One plausible explanation involves sex hormones, which exert differential metabolic and vascular effects. In men, declining testosterone levels with aging are associated with greater central adiposity and less favorable cardiometabolic profiles, which may strengthen the observed associations for IR‐related indices [27]. In women, estrogen has vascular protective effects, and even low residual levels during the peri‐ or postmenopausal period may partially preserve vascular protection, potentially attenuating the association [28]. Differences in fat distribution and baseline cardiometabolic risk may further contribute to heterogeneous associations [29].

From a clinical perspective, TyG and TyG‐BMI can be obtained at low‐cost from routinely collected laboratory and anthropometric measurements. In an EVT‐treated acute BAO cohort, the observed associations with 1‐year disability and death suggest potential utility for early risk stratification and may support closer surveillance and more tailored post‐procedural care. Specifically, patients with elevated TyG or TyG‐BMI may be considered for closer early post‐EVT monitoring, closer cardiometabolic risk‐factor reassessment, and a more structured follow‐up schedule. These indices may also help identify individuals who could benefit from intensified secondary prevention strategies, including optimization of glycemic control, lipid management, blood pressure control, and targeted lifestyle interventions, alongside standard stroke care. Given the modest discrimination, their clinical usefulness is likely greatest when integrated with established clinical and imaging predictors, and future studies should evaluate their incremental value and externally validate multivariable prediction models. TyG‐BMI may also capture an additional metabolic dimension beyond established clinical and imaging predictors, and the signal appeared more pronounced in men. These indices may support risk communication and follow‐up planning and help inform rehabilitation intensity and secondary prevention strategies, pending external validation.

These findings should be interpreted in light of several constraints. The retrospective nature and single‐center setting may have influenced case selection and reduced external applicability. Because the cohort was limited to EVT‐treated patients, generalization to other management strategies is uncertain. Although multivariable adjustment was performed, unmeasured factors may still have contributed to the observed associations. Because covariate selection was partly informed by baseline comparisons (*p* < 0.10), some clinically relevant confounders may not have been included, and residual confounding remains possible. Further confirmation in multicenter prospective cohorts is required, particularly for the sex interaction, together with work to define clinically useful cutoffs and investigate underlying mechanisms.

## 5 Conclusion

Higher TyG and TyG‐BMI were associated with 1‐year unfavorable functional outcome and mortality after EVT for acute BAO. The association between TyG‐BMI and 1‐year mortality appeared to vary by sex, with an association observed in men but not in women. These low‐cost metabolic indices may add complementary information to established clinical and imaging factors for long‐term risk assessment after EVT in BAO.

## Author Contributions

Q.W., H.L., and Y.C. analyzed the data and contributed to the original draft. Q.W., H.L., Y.C., N.L., L.Y., and L.W. collected data. C.W., W.Z., L.J., S.L., Q.M., X.J. contributed to the review and revision of the manuscript. Q.W. and C.L. conceived and designed the study. All authors have read and approved the final manuscript.

## Funding

This work was supported by Beijing High‐level Innovation and Entrepreneurship Talent Support Program (NO. G202522128), Xuanwu Hospital Talent Convergence Program (NO. HZ2025PYDTR011), The Non‐profit Central Research Institute Fund of Chinese Academy of Medical Sciences (NO. 2023‐JKCS‐08), and Beijing Hospitals Authority Youth Programme (NO. QML20230805). The funding agencies had no role in the study design; data collection, analysis, or interpretation; manuscript preparation; or the decision to submit the work for publication.

## Ethics Statement

The study protocol received approval from the Ethics Committee of Xuanwu Hospital, Capital Medical University (Approval No. 2017–030). Written informed consent was obtained from all of the enrolled patients or from their legal representatives (when appropriate), which aligns with the Declaration of Helsinki.

## Conflicts of Interest

The authors declare no conflicts of interest.

## Supporting information


**Figure S1:** Receiver operating characteristic curves for identifying unfavorable outcome and mortality at 1 year according to the TyG and TyG‐BMI indices.
**Figure S2:** Subgroup analyses of the association between TyG index and 1‐year unfavorable outcome.
**Figure S3:** Subgroup analyses of the association between TyG‐BMI and 1‐year unfavorable outcome.
**Figure S4:** Subgroup analyses of the association between TyG index and 1‐year mortality.
**Table S1:** Baseline characteristics of study participants with death and survival at 1 year.
**Table S2:** Incidence of serious adverse events during the hospital stay according to tertiles of the TyG index.
**Table S3:** Incidence of serious adverse events during the hospital stay according to tertiles of the TyG‐BMI.

## Data Availability

Data are available from the corresponding author upon reasonable request.
