# Supplementary material for: Association of the Triglyceride–Glucose (TyG) Index and TyG‐BMI With 1‐Year Outcomes Following Endovascular Treatment in Acute Basilar Artery Occlusion
Source: CNS Neurosci Ther. 2026 Apr 23;32(4):e70898. doi: 10.1002/cns.70898 (PMC13104133; doi:10.1002/cns.70898)
Supplement: Supplementary file 1 — Figure S1: Receiver operating characteristic curves for identifying unfavorable outcome and mortality at 1 year according to the TyG and TyG‐BMI indices. Figure S2: Subgroup analyses of the association between TyG index and 1‐year unfavorable outcome. Figure S3: Subgroup analyses of the association between TyG‐BMI and 1‐year unfavorable outcome. Figure S4: Subgroup analyses of the association between TyG index and 1‐year mortality. Table S1: Baseline characteristics of study participants with death and survival at 1 year. Table S2: Incidence of serious adverse events during the hospital stay according to tertiles of the TyG index. Table S3: Incidence of serious adverse events during the hospital stay according to tertiles of the TyG‐BMI. [file CNS-32-e70898-s001.docx]

FIGURE S1 | Receiver operating characteristic (ROC) curves for identifying unfavorable outcome and mortality at 1 year according to the TyG and TyG-BMI indices.


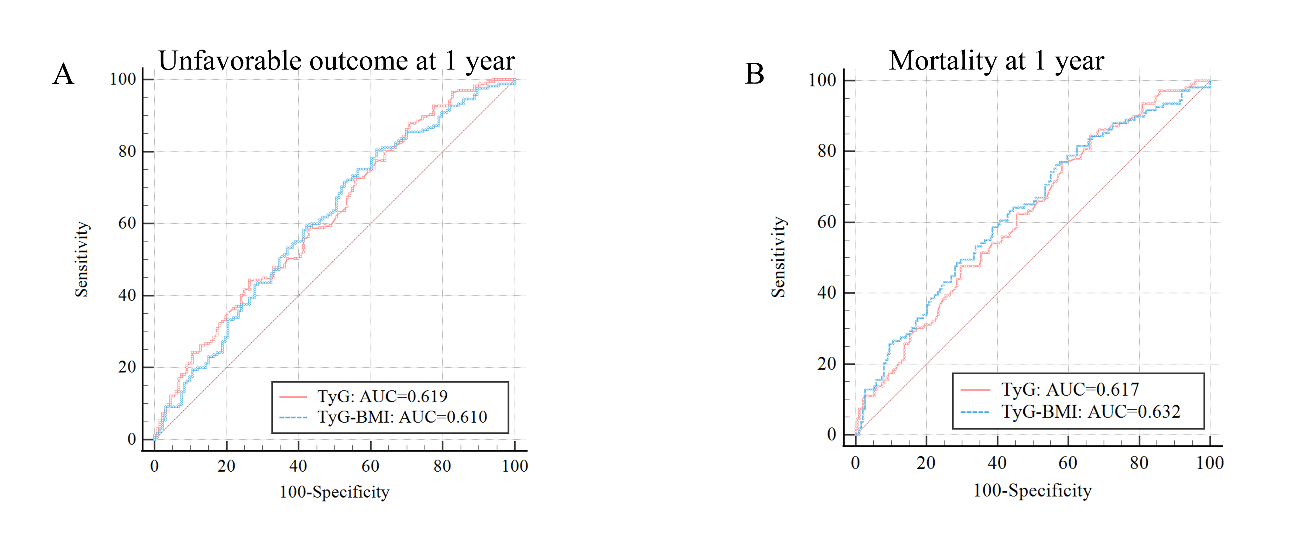


(A) ROC curves for discrimination of 1-year unfavorable functional outcome. (B) ROC curves for discrimination of 1-year all-cause mortality. Areas under the curve (AUCs) were 0.619 for TyG and 0.610 for TyG-BMI for unfavorable outcome (*p* = 0.7098), and 0.617 for TyG and 0.632 for TyG-BMI for mortality (*p* = 0.5790).

AUC, areas under the curve; TyG, triglyceride–glucose index; TyG-BMI, triglyceride–glucose–body mass index.

FIGURE S2 | Subgroup analyses of the association between TyG index and 1-year unfavorable outcome.


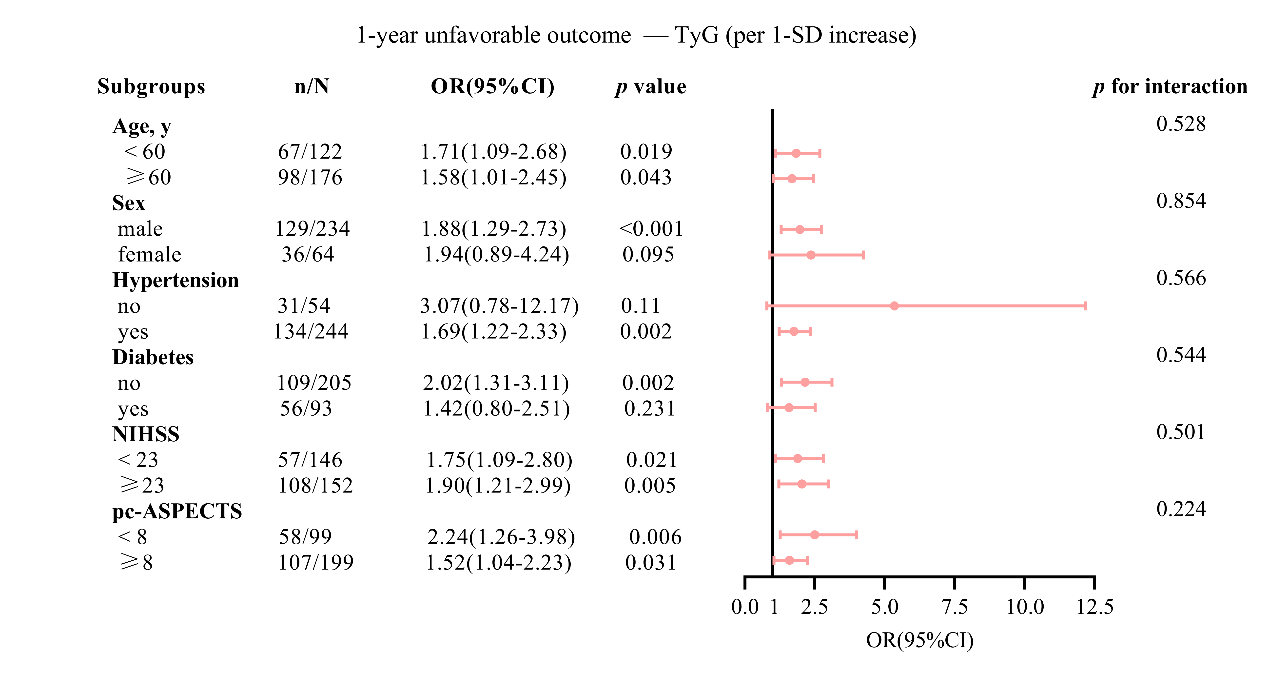


Forest plot of adjusted ORs for 1-year unfavorable outcome per 1-SD increase in TyG index across prespecified subgroups. Points indicate ORs and horizontal lines indicate 95% CIs; the vertical line denotes OR = 1. No significant interactions were detected for prespecified subgroups. Adjustments were consistent with the primary multivariable model (Table 2).

OR, odds ratio, CI, confidence interval; SD, standard deviation; TyG index, triglyceride–glucose index.

FIGURE S3 | Subgroup analyses of the association between TyG-BMI and 1-year unfavorable outcome.


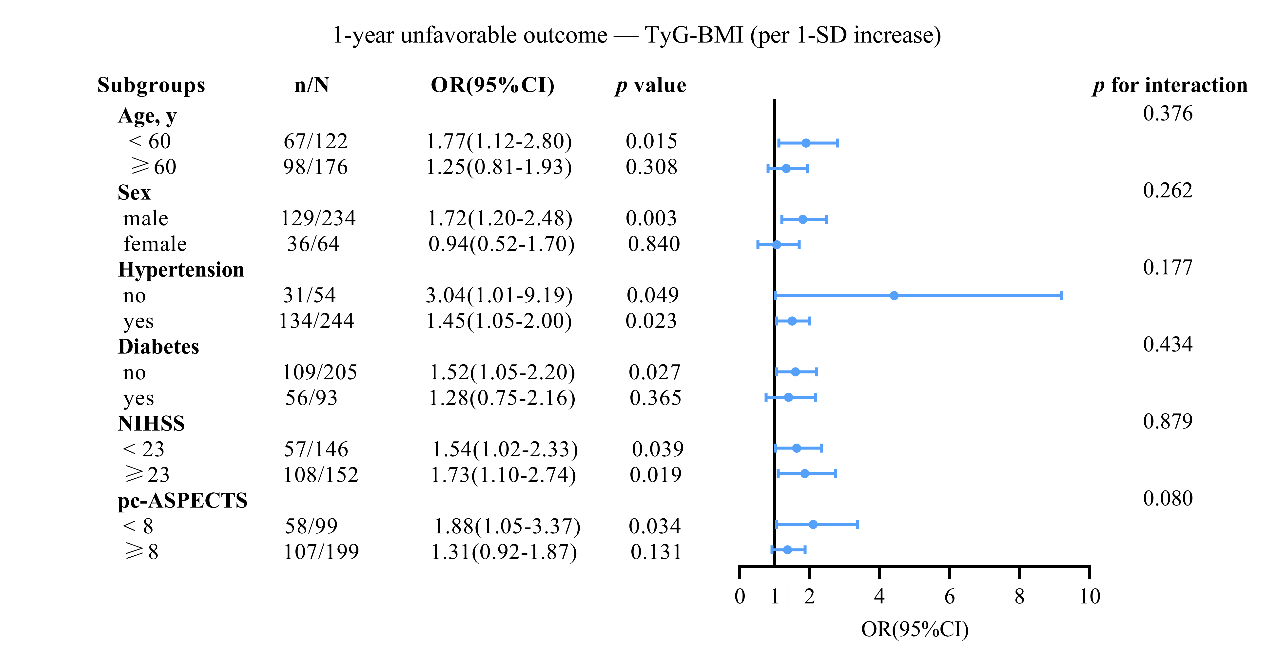


Forest plot of adjusted ORs for 1-year unfavorable outcome per 1-SD increase in TyG- BMI across prespecified subgroups. Points indicate ORs and horizontal lines indicate 95% CIs; the vertical line denotes OR = 1. No significant interactions were detected for prespecified subgroups. Adjustments were consistent with the primary multivariable model (Table 2).

OR, odds ratio, CI, confidence interval; SD, standard deviation; TyG-BMI, triglyceride–glucose-body mass index.

FIGURE S4 | Subgroup analyses of the association between TyG index and 1-year mortality.


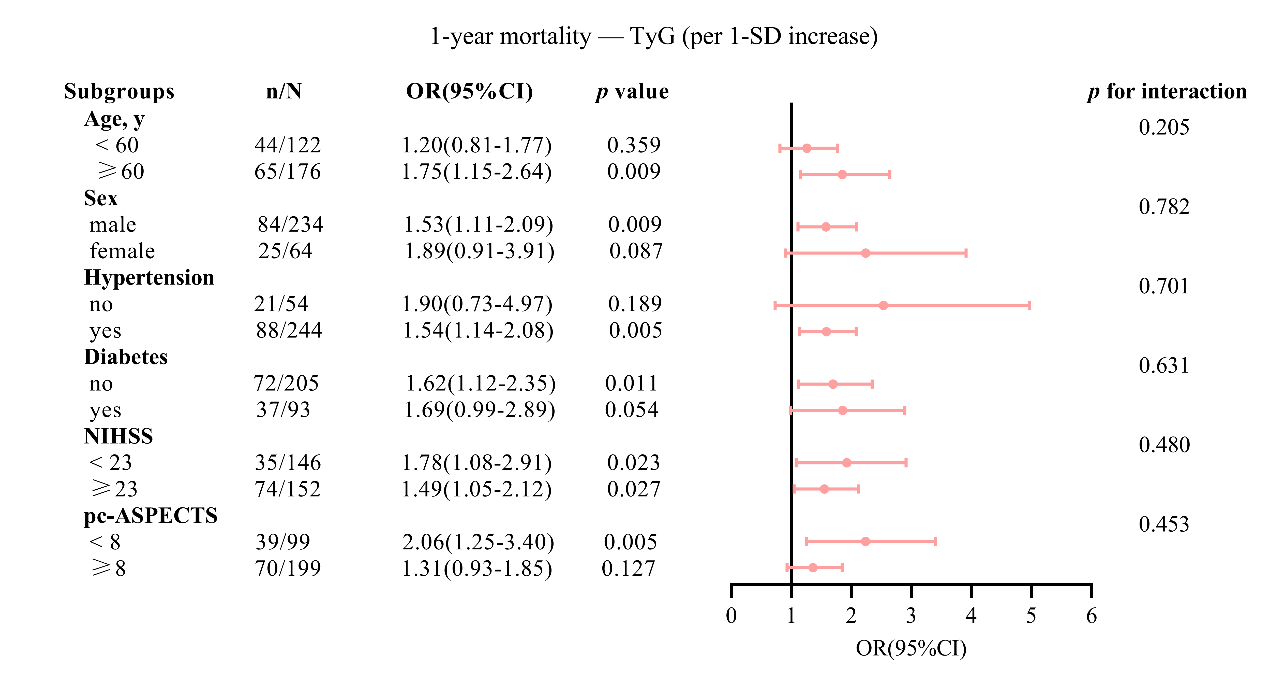


Forest plot of adjusted ORs for 1-year mortality per 1-SD increase in TyG index across prespecified subgroups. Points indicate ORs and horizontal lines indicate 95% CIs; the vertical line denotes OR = 1. No significant interactions were detected for prespecified subgroups. Adjustments were consistent with the primary multivariable model (Table 2).

OR, odds ratio, CI, confidence interval; SD, standard deviation; TyG index, triglyceride–glucose index.

Table S1 Baseline characteristics of study participants with death and survival at 1 year.

| Characteristics | Total sample  (n=298) | | Survivors  (n=189) | | Non-survivors  (n=109) | | *p* value |  |
| --- | --- | --- | --- | --- | --- | --- | --- | --- |
| Demographics | | | | | | | |  |
| Age (years) | 62(55-68) | | 61(55.5-68) | | 62(54-69.5) | | 0.388 |  |
| Male, n (%) | 234(78.5) | | 150(79.4) | | 84(77.1) | | 0.641 |  |
| BMI (kg/m^2^) | 26.02(24.22-27.76) | | 25.71(23.91-27.61) | | 26.99(25.02-28.73) | | 0.003 |  |
| Medical history, n (%) | | | | | | | |  |
| Hypertension | 244(81.9) | | 156(82.5) | | 88(80.7) | | 0.697 |  |
| Diabetes mellitus | 93(31.2) | | 56(29.6) | | 37(33.9) | | 0.439 |  |
| Hyperlipidemia | 118(39.6) | | 75(39.7) | | 43(39.4) | | 0.968 |  |
| Atrial fibrillation | 50(16.8) | | 31(16.4) | | 19(17.4) | | 0.819 |  |
| Coronary heart disease | 51(17.1) | | 30(15.9) | | 21(19.3) | | 0.454 |  |
| Ischemic stroke | 97(32.6) | | 59(31.2) | | 38(34.9) | | 0.518 |  |
| Smoking | 134(45) | | 81(42.9) | | 53(48.6) | | 0.335 |  |
| Drinking | 114(38.3) | | 73(38.6) | | 41(37.6) | | 0.863 |  |
| Use of antiplatelet drugs | | 84(28.2) | | 47(24.9) | | 37(33.9) | | 0.093 |
| Use of anticoagulant drugs | | 10(3.4) | | 9(4.8) | | 1(0.9) | | 0.099 |
| Use of hypolipidemic drugs | | 48(16.1) | | 31(16.4) | | 17(15.6) | | 0.855 |
| Use of hypoglycemic drugs | | 70(23.5) | | 42(22.2) | | 28(25.7) | | 0.497 |
| Use of antihypertensive drugs | | 192(64.4) | | 116(61.4) | | 76(69.7) | | 0.147 |
| NIHSS on admission | | 23(15-34) | | 19(14-30.5) | | 28(21-37.5) | | <0.001 |
| pc-ASPECTS | | 8(7-9.25) | | 8(7-10) | | 8(7-9) | | 0.200 |
| Premorbid mRS | | 0(0-0) | | 0(0-0) | | 0(0-1) | | 0.011 |
| GCS | | 7(4-11) | | 8(5-11) | | 5(3-9) | | <0.001 |
| Blood pressure on admission | | | | | | | | |
| SBP | | 153.56(24.30) | | 152.89(23.68) | | 154.71(25.41) | | 0.536 |
| DBP | | 86(77-96) | | 85(76-95) | | 87(78-98) | | 0.189 |
| Stroke etiology, n (%) | | |  | |  | | 0.718 | |
| Large vessel atherosclerosis | 246(82.6) | | 158(83.6) | | 88(80.7) | |  |  |
| Cardioembolic | 46(15.4) | | 28(14.8) | | 18(16.5) | |  |  |
| Other causes | 6(2.0) | | 3(1.6) | | 3(2.8) | |  |  |
| IVT, n (%) | | 65(21.8) | | 41(21.7) | | 24(22.0) | | 0.948 |
| Tirofiban treatment, n (%) | | 218(73.2) | | 147(77.8) | | 71(65.1) | | 0.018 |
| Time intervals, min | | | | | | | | |
| OTP | 490(365-740) | | 528(371.5-776) | | 465(336-700.5) | | 0.086 |  |
| OTR | 573.5(449-853.5) | | 600(451-877.5) | | 560(430-780) | | 0.228 |  |
| General anesthesia, n (%) | | 150(50.3) | | 89(47.1) | | 61(56) | | 0.140 |
| Interventional procedures, n (%) | | | | | | | | |
| Stent retriever | | 195(65.4) | | 115(60.8) | | 80(73.4) | | 0.028 |
| Aspiration | | 175(58.7) | | 111(58.7) | | 64(58.7) | | 0.998 |
| Intra-arterial thrombolysis | | 44(14.8) | | 27(14.3) | | 17(15.6) | | 0.759 |
| Stenting | | 104(34.9) | | 68(36) | | 36(33) | | 0.607 |
| Balloon angioplasty | | 110(36.9) | | 69(36.5) | | 41(37.6) | | 0.849 |
| mTICI 2b-3, n (%) | | 282(94.6) | | 183(96.8) | | 99(90.8) | | 0.027 |
| Laboratory parameters | | | | | | | | |
| Fasting glucose, mg/dL | 135.78(111.50-184.16) | | 127.40(106.79-160.65) | | 157.31(123.08-217.95) | | <0.001 |  |
| Total cholesterol, mg/dL | 162.41(135.64-188.90) | | 164.87(41.59) | | 168.32(40.28) | | 0.486 |  |
| TG, mg/dL | 100.06(71.5-150.75) | | 97.40(65.52-147.87) | | 108.02(77.48-152.74) | | 0.085 |  |
| TyG | 8.83(8.43-9.40) | | 8.74(8.34-9.29) | | 9.00(8.62-9.66) | | <0.001 |  |
| TyG-BMI | 234.37(39.51) | | 227.78(37.26) | | 245.78(40.84) | | <0.001 |  |

Data were presented as number (%), median (interquartile range) or mean (±standard deviation). BMI, body mass index; NIHSS, National Institutes of Health Stroke Scale; pc-ASPECTS, posterior circulation Alberta Stroke Program Early Computed Tomography Score; GCS, Glasgow Coma Scale; SBP, systolic blood pressure; DBP, diastolic blood pressure; IVT, Intravenous thrombolysis; OTP, onset-to-puncture; OTR, onset-to-reperfusion; mTICI, modified Thrombolysis in Cerebral Infarction; TG, triglycerides; TyG index, triglyceride–glucose index; TyG-BMI, triglyceride–glucose-body mass index.

Table S2 Incidence of serious adverse events during the hospital stay according to the tertiles of TyG index.

| Complication, n (%) | TyG, categorical variable (tertiles) | | | *p* value |
| --- | --- | --- | --- | --- |
|  | Lower | Medium | Upper |  |
| Mortality | 9(9) | 16(16.2) | 15(15.2) | 0.276 |
| sICH | 3(3) | 10(10.1) | 10(10.1) | 0.095 |
| Any ICH | 18(18) | 18(18.2) | 19(19.2) | 0.973 |
| Procedural-related complication | | | | |
| Vessel perforation | 0(0) | 1(1) | 0(0) | 0.365 |
| Vessel dissection | 1(1) | 1(1) | 1(1) | 1 |
| Distal embolization | 2(2) | 0(0) | 0(0) | 0.136 |
| Other serious adverse events | | | | |
| Pneumonia | 77(77) | 69(69.7) | 72(72.7) | 0.505 |
| Brain herniation | 6(6) | 9(9.1) | 14(14.1) | 0.148 |
| Gastrointestinal hemorrhage | 19(19) | 19(19.2) | 21(21.2) | 0.910 |
| Cardiac ischemia | 5(5) | 3(3) | 2(2) | 0.494 |
| Acute heart failure | 3(3) | 4(4) | 0(0) | 0.149 |
| Acute respiratory failure | 19(19) | 18(18.2) | 21(21.2) | 0.856 |

Data were presented as number (%).

sICH, symptomatic intracranial hemorrhage; ICH, intracranial hemorrhage; TyG index, triglyceride–glucose index.

Table S3 Incidence of serious adverse events during the hospital stay according to the tertiles of TyG-BMI.

| Complication, n (%) | TyG-BMI, categorical variable (tertiles) | | | *p* value |
| --- | --- | --- | --- | --- |
|  | Lower | Medium | Upper |  |
| Mortality | 10(10.1) | 11(11.0) | 19(19.2) | 0.118 |
| sICH | 3(3) | 8(8) | 12(12.1) | 0.056 |
| Any ICH | 17(17.2) | 17(17.0) | 21(21.2) | 0.688 |
| Procedural-related complication | | | | |
| Vessel perforation | 0(0) | 1(1.0) | 0(0) | 0.334 |
| Vessel dissection | 0(0) | 2(2.0) | 1(1) | 0.250 |
| Distal embolization | 1(1) | 0(0) | 1(1) | 0.440 |
| Other serious adverse events | | | | |
| Pneumonia | 74(74.7) | 78(78.0) | 66(66.7) | 0.179 |
| Brain herniation | 8(8.1) | 4(4) | 17(17.2) | 0.006 |
| Gastrointestinal hemorrhage | 15(15.2) | 27(27.0) | 17(17.2) | 0.080 |
| Cardiac ischemia | 3(3) | 3(3) | 4(4) | 0.901 |
| Acute heart failure | 2(2) | 3(3) | 2(2) | 0.875 |
| Acute respiratory failure | 16(16.2) | 19(19.0) | 23(23.2) | 0.449 |

Data were presented as number (%).

sICH, symptomatic intracranial hemorrhage; ICH, intracranial hemorrhage; TyG-BMI, triglyceride–glucose-body mass index.
